# No evidence for male attraction to cephalic labial gland pheromones in *Bombus terrestris* (Hymenoptera: Apidae)

**DOI:** 10.1093/jisesa/ieag041

**Published:** 2026-05-21

**Authors:** Danielle Claudia Allam, Manuela Carnaghi, G Mandela Fernández-Grandon, Marina P Arbetman, Mark J F Brown

**Affiliations:** Department of Biological Sciences, Royal Holloway University of London, Egham, UK; Natural Resources Institute, University of Greenwich, Kent, UK; Natural Resources Institute, University of Greenwich, Kent, UK; Instituto de Investigaciones en Biodiversidad y Medioambiente (INIBIOMA), Universidad Nacional del Comahue and CONICET, Bariloche, Argentina; Department of Biological Sciences, Royal Holloway University of London, Egham, UK; Department of Zoology, University of Cambridge, Cambridge, UK

**Keywords:** invasive pollinator, olfactory behavior, chemical ecology, reproductive communication, Y-tube olfactometer

## Abstract

The ecological impact of *Bombus terrestris* (Linnaeus, 1758) (Hymenoptera: Apidae) following its introduction to regions outside its native range has raised significant concerns, particularly regarding the decline of native pollinators. Male *B. terrestris* secrete pheromones that are used in reproductive communication, and which have the potential to be used as trap attractants to control invasive populations. However, while gynes (virgin reproductive females) are known to be attracted to these pheromones, nothing is known about behavioral responses of male *B. terrestris* to them. Here, we investigated whether male *B. terrestris* exhibit attraction to pheromone extracts from conspecific males under controlled laboratory conditions. A Y-tube olfactometer was used to present *B. terrestris* males with a choice between a pheromone extract and a control treatment. Males were shown to respond to a positive control of lavender oil; however, they exhibited no attraction to the pheromone extracts. Several factors, such as pheromone concentration, age of individuals, or apparatus design, may have influenced these results. These findings contribute to our knowledge of bumblebee chemical communication and may inform future experimental design to assess pheromone attraction in bumblebees.

## Introduction

The introduction of *Bombus terrestris* (Linnaeus, 1758) (Hymenoptera: Apidae) to regions outside its native range has had significant ecological consequences, such as in South America, where it has contributed to the rapid decline of native pollinators like *Bombus dahlbomii* (Guérin-Méneville, 1835) (Hymenoptera: Apidae) (Arbetman et al. 2013, Morales et al. 2013). Controlling invasive populations is key to mitigating their negative ecological impact, but as of yet, there are no effective ways to selectively control invasive bumblebees. Pest insects in agricultural systems are often monitored and controlled using pheromone traps or lures (Svensson et al. 2012, Klassen et al. 2023), and bumblebees have been found as bycatch in these systems (Grocock et al. 2020, Akotsen-Mensah et al. 2025). This suggests that a similar approach has a potential to control invasive bumblebee populations, like *B. terrestris* in South America, without jeopardizing an endangered native bumblebee.

Pheromone communication is a fundamental aspect of bumblebee reproductive behavior. Male bumblebees, including those of *B. terrestris*, produce pheromones in the cephalic part of their labial gland (CLG) (Agren et al. 1979). *Bombus terrestris* males, like many other bumblebee species, employ a patrolling pre-mating strategy in which they repeatedly fly along established routes and deposit CLG pheromones in specific spots (scent marking) to attract conspecific virgin queens for mating (Kullenberg et al. 1970, Goulson 2010, Valterová et al. 2019) Multiple males will share a flight route and scent-marking spots, as first shown by Darwin (Freeman 1968). Kubo et al. (2017) demonstrated that male *Bombus ardens ardens* (Smith 1879) are attracted to conspecific male pheromones, suggesting that CLG pheromones are used by males when sharing flight routes and scent-marking spots. The chemical composition of pheromones is species-specific, with variation even within populations and across ages (Žáček et al. 2009, Coppée et al. 2011, Lecocq et al. 2015, Valterová et al. 2019). This ensures reproductive isolation even when different species occupy the same habitat (Lecocq et al. 2015, Valterová et al. 2019). The pheromone production and chemical composition of *B. terrestris* is highly age-dependent (Žáček et al. 2009), consequently, understanding the role of these pheromones is important for applied research, such as developing pheromone-based control strategies for invasive species.

*Bombus terrestris* males are capable of multiple matings and mate readily in laboratory settings (Tasei et al. 1998, Amin et al. 2012, Gosterit and Gurel 2016), which indicates that this species is responsive to reproductive cues in laboratory conditions. Y-tube olfactometers, which provide a controlled airflow to isolate olfactory-driven behavioral responses in laboratory settings, have been instrumental in advancing our understanding of bumblebee mating pheromone detection (Kubo et al. 2017). Previous studies have used both arena assays and Y-tube olfactometers to investigate behavioural responses of bumblebees to male pheromones (Coppée et al. 2011, Lecocq et al. 2015, Kubo et al. 2017). Here, we used a Y-tube olfactometer to investigate whether male *B. terrestris* exhibit attraction to conspecific CLG pheromone extracts. Although *B. terrestris* (subgenus *Bombus sensu stricto*) and *B. ardens ardens* (subgenus *Pyrobombus*) are phylogenetically distant and may therefore differ in their pheromone composition and mating behavior, the experimental framework on *B. ardens ardens* established by Kubo et al. (2017) was adopted as a methodological model. In addition to its relevance for control and management of this invasive species, *B. terrestris’*s experimental amenability makes it an excellent system for testing whether male-to-male pheromone attraction is a trait conserved across different subgenera. Testing established behavioral assays across different species is a common comparative approach that can be used to determine if specific mechanisms are shared across taxa (Mundinger et al. 2025). By applying this methodology to *B. terrestris*, we aimed to evaluate whether male-to-male pheromone attraction is a shared trait or varies between subgenera. The setup was validated using lavender oil, a well-documented attractant for bumblebees (Balfour et al. 2013, Roy et al. 2016, Valchev et al. 2022). By eliminating environmental variables and isolating olfactory-driven behavior of *B. terrestris* males to the pheromone, this study contributes to our understanding of chemical communication in bumblebees, specifically males, and informs the potential application of pheromone-based strategies for managing invasive bumblebee populations.

## Methods

### CLG Pheromone Extraction

Six colonies of *B. terrestris* were obtained from Biobest (Westerlo, Belgium) and were maintained at 25 °C, 50% humidity, with *ad libitum* pollen and 50% (w/v) sugar–water solution. Within these colonies, newly emerged males were collected, labelled, and reared individually (under these conditions) until used for pheromone extraction at 7 d old, following Kubo et al. (2017). Dissection of the CLGs and pheromone extraction was done following the methodology in Terzo et al. (2005), Terzo et al. (2007), and De Meulemeester et al. (2011), with glands placed in 200 µl of hexane at 20 °C for 24 h. Extracts were stored at −80 °C until used in behavioral assays.

### Y-Tube Assay Setup

**Fig. 1.**
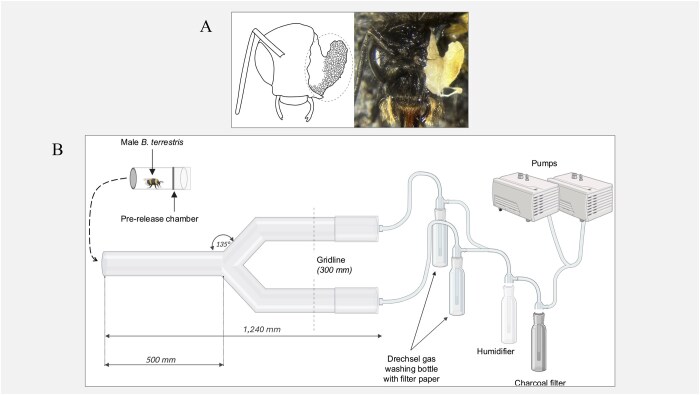
A) Diagram (left) and photograph (right) of the cephalic part of the labial gland in a male *B.terrestris*. The gland is indicated by the dotted circle. B) Diagram of the Y-tube olfactometer setup, with 1 main arm (500 mm length, 70 mm diameter) and 2 diverging arms of the same dimensions, with a joint length of 240 mm, resulting in a total length of 1,240 mm. The airflow system is fed through an activated charcoal filter, then humidified, delivering air at 2.5 ml/min to each arm. Silicone tubing connects the pumps to the Drechsel glass wash bottles and then arms.

The experimental setup utilized apparatus adapted from Fernández-Grandon et al. (2015) (Figure 1), while the experimental protocol was based on the methodology outlined by Kubo et al. (2017). The Y-tube olfactometer consisted of 1 main arm (500 mm length, 70 mm diameter) and 2 diverging arms (500 mm length, 70 mm diameter), each connected to the odor delivery chamber, that is, a Drechsel gas washing bottle, via silicone tubing. The joint connecting the arms was 20 mm, resulting in a total length of 1,240 mm (Figure 1).

To obtain the large quantities required for behavioral assays, test males were obtained from Biobest (Westerlo, Belgium) as “masculino” (male-only) boxes. Because the source colonies were unknown, relatedness among test individuals and their relationship to bees used for pheromone extraction could not be determined. To reduce age-related variation, since precise ages at arrival were unknown, assays were performed within the first 3 d after receipt, although individual ages remained uncertain. Before use, individuals were visually assessed for their hair quality and quantity, which is an indicator of age, and only the freshest individuals were selected. They were housed in wooden boxes at 25 °C, 40% relative humidity, with sugar water and pollen *ad libitum*.

At the start of each day, the Y-tube and associated components were cleaned with 70% ethanol, and pumps were run for 10 min with clean air to remove any residual odors. Air was supplied to the apparatus at 2.5 ml/min by 2 Fisher pumps (Fisher Scientific, United Kingdom) (model number FB70155/EUR), after being passed through an activated charcoal filter and then humidified.

### Experimental Procedure

Assays were conducted under red light to eliminate visual interference, and laboratory conditions were maintained at 25 °C and 40% RH. For each trial, a filter paper treated with 0.4 μl pheromone extract was placed into one odor delivery chamber, and a second filter paper with 0.4 μl hexane (control) in the other. Filter papers were replaced after every trial. Volumes matched the dilution ratio used by Kubo et al. (2017).

Males were placed individually in the pre-release chamber at the base of the Y-tube, and after one minute of acclimation time, the bee was released into the main arm. A choice was recorded when a male crossed a grid line located 300 mm into one of the arms. The bee was given 5 min to make a choice, and if there was no choice within this time, the male was excluded from the analysis.

Positive controls using lavender oil and arm bias controls using hexane on both sides were also conducted throughout to confirm correct apparatus function. The lavender oil concentration used was 10^−4^ dilution (0.01%) (Habitual Pure Lavender Essential Oil, United Kingdom). As further control, the arm containing the treatment was alternated every 5 trials, and each bee was only used once.

Choice data were analysed with chi-squared tests performed using RStudio (version 4.4.1) (Posit team 2024).

This research complied with the ethical requirements for animal experimentation in the United Kingdom and was approved by the Royal Holloway Research Ethics Committee.

## Results

**Fig. 2.**
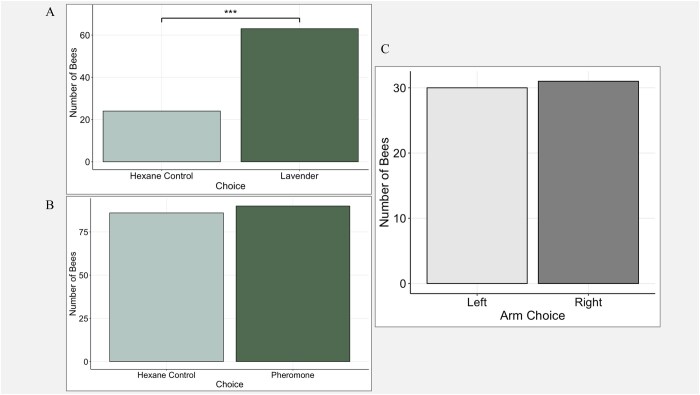
Choice responses of *B. terrestris* males in Y-tube olfactometer assays. A) Number of choices made to the arm containing lavender versus the hexane control arm. B) Number of choices made to the arm containing the pheromone extract versus the hexane control. C) Number of choices made to the left versus right arm when both arms contained only hexane. For all panels, each choice represents an individual *B. terrestris* male. Bars represent the total number of individuals choosing each option. Statistical significance was determined using a Chi-squared test, and significant differences are indicated by horizontal bars with asterisks (***P < 0.001).

In the lavender trials, of the 87 males tested, 87 made a choice. Males exhibited a significant preference for the lavender-treated arm over the hexane-treated control arm (χ^2^_(1)_ = 17.483, *P* = 2.899e–05; Figure 2), confirming that the apparatus detected olfactory preferences of *B. terrestris* males. In the arm-bias trials, of the 61 males tested, 61 made a choice. No directional bias was observed when both arms contained hexane (χ^2^_(1)_ = 0.0164, *P* = 0.8981; Figure 2).

For pheromone trials, of the 182 males tested, 176 made a choice. There was no significant preference for the pheromone-treated arm over the hexane control arm (χ^2^_(1)_ = 0.091, *P* = 0.763; Figure 2); thus, no attraction to conspecific pheromone extracts by *B. terrestris* males was detected under the tested laboratory conditions.

We also examined daily variation across the experimental trials. We found no significant arm bias on any test day (mean left arm choice: 49.5%; all *P* > 0.05; Table 1). However, variation was observed in the attractiveness of lavender-treated discs compared to control discs. On several days (3 July, 4 July, and 22 July), bees showed a significant preference for lavender-treated discs. (Table 1). On other days, no significant preference for lavender-treated discs was observed (Table 1).

**Table 1.** Daily behavioral responses of *B. terrestris* males across 3 trial types: arm bias (control), lavender (positive control), and CLG pheromone extract

	Arm bias				Lavender				Pheromone			
Date	Left	Right	% left	*P* value	Lavender	Control	% Lavender	*P* value	Pheromone	Control	% Pheromone	*P* value
**1 July**	2	3	40.0	1.00	8	3	72.7	0.09	–	–	–	–
**3 July**	2	3	40.0	1.00	12	3	80.0	0.003	–	–	–	–
**4 July**	3	2	60.0	1.00	7	0	100.0	0.001	5	5	50.0	1.00
**5 July**	2	4	33.3	0.57	–	–	–	–	3	1	75.0	0.49
**16 July**	7	8	46.7	1.00	–	–	–	–	5	17	22.7	0.001
**17 July**	3	4	42.9	1.00	6	3	66.7	0.35	18	11	62.1	0.11
**21 July**	5	3	62.5	0.62	8	5	61.5	0.43	13	17	43.3	0.44
**22 July**	3	2	60.0	1.00	5	0	100.0	0.01	22	15	59.5	0.16
**23 July**	3	2	60.0	1.00	4	3	57.1	1.00	16	14	53.3	0.80
**24 July**	–	–	–	–	13	7	65.0	0.11	8	6	57.1	0.71
**Total**	**30**	**31**	**49.2**		**63**	**24**	**72.4**		**90**	**86**	**51.1**	

For each test and day, the number of bees choosing each arm, the proportion choosing the treatment arm (or left arm in arm bias), and the *P*-values from Fisher’s Exact Test are shown. Tests were conducted from 1 July 2025 to 24 July 2025.

Pheromone-treated discs elicited more variable responses. On 16 July, bees showed a significant preference for the control discs over the pheromone-treated discs, with only 22.7% of bees choosing the pheromone (Table 1). On other days, the proportion of bees choosing the pheromone ranged from 43.3% to 75.0%, but none of these differences was statistically significant (Table 1).

## Discussion

*Bombus terrestris* males exhibited a strong and statistically significant preference for the lavender-treated arm in the Y-tube olfactometer, confirming the apparatus’ reliability in detecting olfactory attraction under laboratory conditions. Additionally, no directional bias was observed in hexane-only trials, further validating the reliability of the Y-tube olfactometer. This approach is consistent with previous studies using positive or negative controls to validate behavioral responsiveness and apparatus reliability (Boer and Duchateau 2006, Bunk et al. 2010, Ceuppens et al. 2015, Kubo et al. 2017).

When presented with a choice between conspecific CLG extracts and a hexane control, *B. terrestris* males showed no significant preference for the extract. This finding suggests that, under the tested laboratory conditions, pheromone extracts do not elicit strong attraction in *B. terrestris* males. This result contrasts with Kubo et al. (2017), where *B. ardens ardens* males and queens were attracted to conspecific pheromone extracts in similar Y-tube assays. This contrast may be attributed to the different chemical profiles of the pheromones between these 2 species. As shown by Žáček et al. (2009) and Valterová et al. (2019), bumblebee pheromone compositions differ greatly between species, so the active components in 1 species may have entirely different sensitivity thresholds than those in another species. Although extracts from conspecific males were used in both studies, the chemical variation between *B. terrestris* and the model species in Kubo et al. (2017), *B. ardens ardens*, may have resulted in the use of a pheromone concentration that was not effective for *B. terrestris*. *Bombus terrestris* may require a different pheromone concentration for attraction, and the concentration tested may have been too high or too low to elicit a response. An obvious alternative explanation is that *B. terrestris* males are not attracted to CLG pheromones. Future studies should test a range of pheromone concentrations to determine the optimal concentration for attraction in *B. terrestris*, if one exists, while keeping all other experimental factors consistent.

Another factor which may have influenced the composition of the pheromone extract is the age of the males used for extraction. While Kubo et al. (2017) demonstrated that *B. ardens ardens* responded to male pheromone extracts using 7-d-old males for the behavioral tests, the specific age of the males used for pheromone extraction was not specified in their study. Regarding *B. terrestris*, Coppée et al. (2011) showed that the attractiveness of CLG secretions to gynes is highly age-dependent, with extracts from 10-d-old males being the most attractive in their study. However, to align with the methodology described in Kubo et al. (2017), we used 7-d-old males for CLG extractions. The decision to use this age group may have resulted in a suboptimal pheromone composition for *B. terrestris*. On the other hand, the use of 7-d-old males for extractions is supported by Šobotník et al. (2008), who noted that *B. terrestris* labial gland secretion reaches a maximum at 6 d old until 10 d old, and by Žáček et al. (2009), who reported that the most abundant EAD-active component, 2,3-dihydrofarnesol, peaks in concentration on days 6 and 7, and then decreases dramatically. Collectively, these results suggest that the extraction timing chosen in this study aligned with the biologically optimal phase of pheromone production in *B. terrestris*, thus validating our methodology.

Furthermore, male *B. terrestris* typically reach sexual maturity and peak mating receptivity between 6 and 12 d post-eclosion, characterized by maximum sperm production and the shortest mating latencies (Tasei et al. 1998, Amin et al. 2012). While the window for peak mating receptivity is relatively early, males can remain reproductively viable for an extended period, with first matings observed as late as 27 d of age (Tasei et al. 1998). Although the exact age of our commercially sourced test males was unknown, the freshest were selected and utilized shortly after arrival to ensure they remained within the window of highest responsiveness.

Additionally, the dimensions of the Y-tube olfactometer used in this study were considerably larger than those used in other bumblebee studies (Boer and Duchateau 2006, Kubo and Ono 2014, Ceuppens et al. 2015, Kubo et al. 2017, Zhang et al. 2020, Salman et al. 2022). While we used a similar airflow rate at the point of entry, and a similar pheromone concentration to Kubo et al. (2017), and even though the setup was validated with lavender as a positive control, further studies using different behavioural chambers, including Y-tubes of different dimensions and flying arenas are recommended to better understand the response of *B. terrestris* to conspecific pheromones.

In conclusion, our findings suggest that, at least under the tested conditions, conspecific CLG extracts may not influence male–male attraction in *B. terrestris*, in contrast to other *Bombus* species (Kubo et al. 2017). Future studies should test the effect of factors such as concentration of the extract, the age of both the bees used for the extract as well as the test males, and the physical dimensions of the Y-tube apparatus. Addressing these will help clarify if *B. terrestris* males are, in fact, attracted to conspecific pheromone extract, and improve the design of future experiments investigating pheromone-based attraction in bumblebees.
